# Rising Incidence of Mucosal Melanoma of the Head and Neck in the United States

**DOI:** 10.1155/2012/231693

**Published:** 2012-12-02

**Authors:** David M. Marcus, Rebecca P. Marcus, Roshan S. Prabhu, Taofeek K. Owonikoko, David H. Lawson, Jeffrey Switchenko, Jonathan J. Beitler

**Affiliations:** ^1^Department of Radiation Oncology, Emory University, 1365 Clifton Road NE, Suite AT225, Atlanta, GA 30322, USA; ^2^Winship Cancer Institute, Emory University, Atlanta, GA, USA; ^3^Department of Dermatology, Columbia University, New York, NY, USA; ^4^Department of Hematology/Oncology, Emory University, Atlanta, GA, USA; ^5^Department of Biostatistics and Bioinformatics, Emory University, Atlanta, GA, USA; ^6^Department of Otolaryngology, Emory University, Atlanta, GA, USA

## Abstract

*Background*. While it is established that the incidence of cutaneous melanoma has risen over time in the United States, the incidence trend for mucosal melanoma of the head and neck (MMHN) is unknown. *Methods*. We used the Surveillance, Epidemiology, and End Results (SEER) database to determine incidence trends for MMHN from 1987 to 2009 in the United States. We determined annual percent change (APC) by weighted least squares and joinpoint regression analysis. *Results*. MMHN incidence increased from 1987 to 2009 (APC 2.4%; *P* < 0.01). Nasal cavity lesions increased in incidence (APC 2.7%; *P* < 0.01) over this duration, while the incidence of non-nasal cavity lesions remained stable. The highest rate of increase was in white females ages 55 to 84 (APC 5.1%; *P* = 0.01). *Conclusions*. The incidence of MMHN in the United States has been rising since 1987. This trend is driven primarily by increased incidence of nasal cavity melanomas.

## 1. Introduction

Malignant melanoma is a relatively common malignancy in the United States and is responsible for the majority of skin cancer deaths. Approximately 70,230 cases of melanoma were diagnosed, and approximately 8,790 people died from melanoma in the United States in 2011 [1]. The incidence of cutaneous melanoma in the United States has been steadily rising since 1975 [2, 3], with the most prominent risk factor being sun exposure [4]. Mucosal melanoma of the head and neck (MMHN) is a rare and aggressive disease that makes up less than 1% of all melanoma cases in the United States [5, 6]. To date, it is unknown whether the incidence of MMHN, which occurs outside of sun exposed areas, has followed the same incidence trend over time as cutaneous melanoma. In our study, we describe trends in incidence rates (IRs) for MMHN in the United States using the National Cancer Institute's Surveillance, Epidemiology, and End Results (SEER) database.

## 2. Materials and Methods

We identified patients using the SEER 9 registries, which encompass nine geographic regions in the United States (Atlanta, Connecticut, Detroit, Hawaii, Iowa, New Mexico, San Francisco-Oakland, Seattle-Puget Sound, and Utah) and include approximately 9.5% of the US population. The SEER 9 population accurately represents the US population in most regards [2]. The SEER 9 registries were chosen for this purpose because each of the member registries has been continuously active throughout the duration of the study period. Using SEER∗Stat Version 7.0.9, we identified patients within the SEER 9 registries with International Classification of Diseases in Oncology (ICD-0–3) diagnosis codes for all histologic variants of melanoma (8720/3–8723/3, 8730/3, 8740/3–8746/3, 8761/3, 8770/3–8772/3) [7]. We included any of the above histologies associated with topography codes for mucosal lip (C00.3–C00.8), tongue (C01.9–C02.9), salivary glands (C07.9–C08.9), floor of mouth (C04.0–C04.9), gums (C03.0–C03.9), other mouth (C05.0–C06.9), nasopharynx (C11.0–C11.9), tonsils (C09.0–C09.9), oropharynx (C10.0–C10.9), hypopharynx (C12.9–C13.9), other pharynx (C14.0–C14.8), nasal cavity (C30.0), middle ear (C30.1), paranasal sinuses (C31.0–C31.9), or larynx (C32.0–C32.9). Cases that were designated as “autopsy or death certificate only” were excluded. 

All data was obtained using SEER∗Stat version 7.0.9, and statistical analysis was performed with SPSS Version 19 (IBM Corporation, Armonk, NY, USA), and Joinpoint Regression Program version 3.5.3 [8]. Our primary endpoints were the percent change (PC) and the annual percent change (APC) in the age-adjusted IR of MMHN from 1987 to 2009, and we evaluated incidence trends for several subgroups according to gender, race, age, and anatomic subsite by a stratified analysis. While incidence data is available for MMHN going back to 1973, there were not enough cases from 1973 to 1986 to perform a meaningful incidence trend analysis. Therefore, we made the decision to limit our analysis of incidence trends to the time period from 1987 to 2009. Incidence was defined as the number of new cancers diagnosed per year, and all IRs represent the number of cases per 1,000,000 persons per year. Incidence was standardized to the United States population for the year 2000. The PC represents the age-adjusted IR in 2009 divided by the age-adjusted IR in 1987. The APC is estimated by weighted-least squares regression to the natural logarithm of age-adjusted incidence rates by year. APCs are presented with their associated *P* values (representing the likelihood that the APC is not significantly different from zero). A *P* value of <0.05 was considered statistically significant. The statistical methods employed in this analysis were similar to those used in prior studies that have reported on trends in cancer incidence [9, 10].

## 3. Results

### 3.1. Patient Characteristics

We identified 452 cases of MMHN in the SEER 9 registry between 1987 and 2009. Of these patients, 237 (52.4%) were female, and 215 (47.6%) were male. The cohort included 383 white patients (84.7%), 24 black patients (5.3%), 44 patients (9.7%) classified as “other” (including American Indian/Alaska Native and Asian/Pacific Islander), and one patient (0.2%) with unknown race. By comparison, the racial distribution of the general US population is 75.1% white, 12.3% black, and 12.5% other races (including American Indian/Alaska Native, Asian, Hawaiian/Pacific Islander, other race, and two or more races) [11]. The most common site of disease was the nasal cavity, comprising 237 patients (52.4%), and 328 patients (72.6%) had disease in a sinonasal location. The majority of patients (56.4%) were age 70 or older. Patient characteristics are illustrated in Table 1. 

### 3.2. Incidence

**Figure 1:**
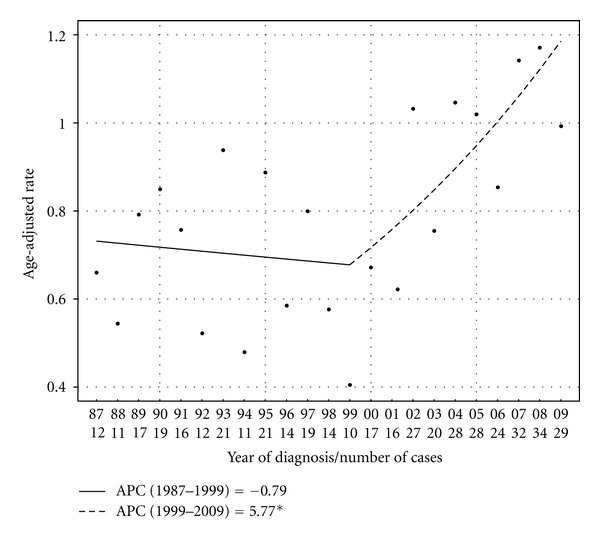
Age-adjusted incidence rates (in cases per million persons per year) with Joinpoint regression trendlines for all patients with MMHN in SEER 9 registries from 1987 to 2009. The APC over this time period was 2.4%.

The age-adjusted incidence of MMHN in the United States has increased over time. From 1987 to 2009, the total PC in the age-adjusted IR for all patients with MMHN was 50.0%, and the APC was 2.4% (*P* < 0.01). Joinpoint analysis demonstrates that this trend was most pronounced from 1999 to 2009, with an APC of 5.8% over that time period, as illustrated in Figure 1.

**Figure 2:**
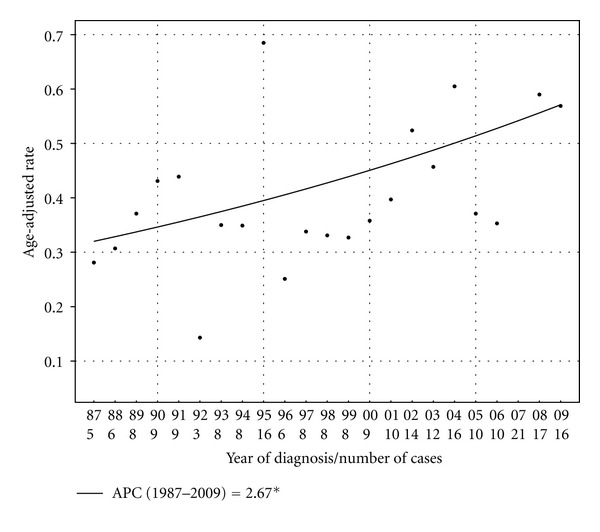
Age-adjusted incidence rates (in cases per million persons per year) with Joinpoint regression trendline for patients with melanoma of the nasal cavity in SEER 9 registries from 1987 to 2009. The APC over this time period was 2.7%.

Details of the total PC and APC in age-adjusted IRs for MMHN by subgroup from 1987 to 2009 are shown in Table 2. By subsite, the rate increase was most pronounced in nasal cavity lesions, with a total PC of 102.9% and APC of 2.7% over the study period (*P* < 0.01), as illustrated in Figure 2. No statistically significant joinpoints were identified for this group. By contrast, there was no statistically significant change in the age-adjusted IR over time for lesions outside of the nasal cavity. Stratified by gender, there was a significant increase in the age-adjusted IR over time for female patients, with a total PC of 45.5% and an APC of 3.4% (*P* < 0.01), while there was no significant change in incidence for male patients over the same time period (PC 35.2%, APC 1.0%, *P* = 0.30). There was increased incidence in white patients, with a total PC of 52.5% and APC of 2.2% (*P* = 0.01); however, due to low patient numbers, incidence trends for other racial groups were unable to be calculated. For white females with nasal cavity lesions, the total PC and APC were 50.4% and 3.5%, respectively (*P* = 0.01). 

**Figure 3:**
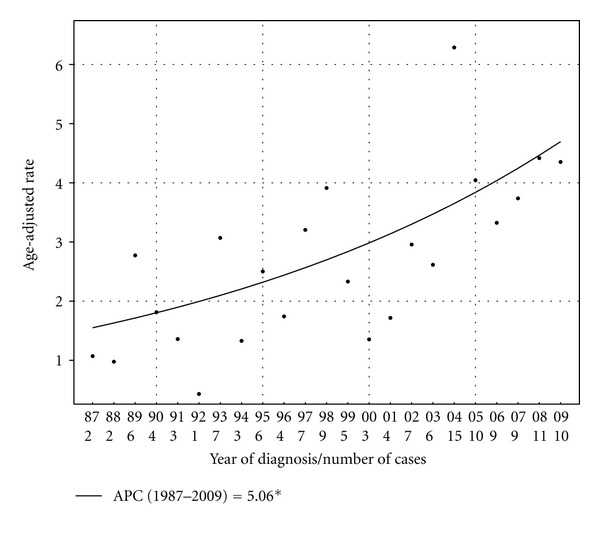
Age-adjusted incidence rates (in cases per million persons per year) with Joinpoint regression trendline for white female patients ages 55 to 84 with MMHN in SEER 9 registries from 1987 to 2009. The APC over this time period was 5.1%.

There were also differences in age-adjusted IR trends between various age groups. For patients of ages 55 to 84 there was a significant trend demonstrating higher age-adjusted IRs over time, with a total PC of 184.4% and an APC of 2.9% from 1987 to 2009. For patients ages 20–54 and ≥85, there were insufficient patient numbers to determine an APC; however, the PC in age-adjusted IRs between 1987 and 2009 for these subgroups were −42.1% and −34.4%, respectively. The specific subset of patients that had the highest total PC and APC in the age-adjusted IR for MMHN from 1987 to 2009 were white females ages 55 to 84. In this cohort, the total PC in age-adjusted incidence of MMHN was 306.3%, and the APC was 5.1% over the study period (*P* < 0.01). No statistically significant joinpoint could be identified for this group. Age-adjusted IRs for white females ages 55 to 84 with MMHN by year are illustrated in Figure 3. 

## 4. Discussion

MMHN is a rare malignancy that makes up less than one percent of all melanomas. Outcomes for patients with MMHN are generally poor, with reported five-year overall survival (OS) rates for all stages ranging from 20 to 48% [12–16]. Sinonasal lesions often present with epistaxis and nasal obstruction, and oral cavity lesions typically present as flat pigmented lesions that may be found on dental examination [17]. There is no firm consensus regarding the optimal management of these tumors; however, the usual primary treatment is surgical resection. Postoperative radiotherapy has been shown to improve local control but has no demonstrated impact on survival [15, 16, 18–20]. Because of the rarity of MMHN, the majority of studies on this disease are single institution reports with limited patient numbers, and reliable data regarding both outcomes and epidemiology are sparse. 

It is firmly established that the incidence of cutaneous melanoma is increasing over time [21, 22]. The 2000 joint annual report on cancer reported that the incidence of cutaneous melanoma in the United States increased by approximately 2.6% per year from 1991 to 1997 [3]. More recently, Simard et al. demonstrated that the APC in the age-adjusted IR for cutaneous melanoma was 2.1% per year for white men and 2.4% per year for white women from 1999 to 2009 in the United States, with subset analysis demonstrating that these trends were mainly due to increasing incidence in men aged older than 55 years and women of all ages [22]. This trend may be due to changes in sun exposure patterns; however, it is also possible that improved detection has played a role. In contrast to the well-documented rise in incidence for cutaneous melanoma, however, there has been no published data to date describing the incidence trend for MMHN. Our analysis of the SEER data demonstrates that the incidence of MMHN in the United States has been rising steadily from 1987 to 2009, with an APC (2.4%) similar to that of cutaneous melanoma over a similar time period. Furthermore, Joinpoint regression analysis demonstrates that the rise in incidence of MMHN was particularly prominent from 1999 to 2009, with an APC of 5.8% over this time period. This trend is driven by an increased incidence of nasal cavity lesions. There is a particularly notable increase in MMHN in white females, with the highest rates of increase observed in white females ages 55 to 84. These findings suggest that there are dynamic etiologic factors that are causing a change in the patterns of presentation in this disease. However, it is unclear what specific risk factors are contributing to the particularly rapid increase in MMHN incidence in this subgroup, and further study is needed to better understand this trend.

Risk factors for the development of cutaneous melanoma are well understood and include exposure to ultraviolet radiation, family history of melanoma, and multiple benign or atypical nevi [23]. Melanocytes in the mucosal layers of the head and neck are thought to be histologically identical to melanocytes in the skin [17]; however, the pathogenesis of MMHN is largely unknown. Several etiologic agents for MMHN have been proposed, including tobacco exposure, occupational exposure to formaldehyde [24], and infection with the human papilloma virus (HPV), none of which are thought to be important risk factors for the development of cutaneous melanoma [25]. Studies have shown an association with mutations in the kit proto-oncogene, which encodes the protein c-kit and is not typically mutated in cutaneous melanoma [26]. Given that cutaneous melanoma and MMHN do not appear to share etiologic factors, it is of interest that the incidence rates of these diseases are rising at similar rates. Further study is needed to better understand the pathogenic basis of these findings.

A possible explanation for our findings is that advances in endoscopy and imaging may have improved detection of this disease over the last 25 years. However, given the infrequency of screening mucosal surfaces of the head and neck in asymptomatic patients and the inherent difficulty in identifying many of these lesions on physical exam, it is unlikely that improved screening would be singularly responsible for such a pronounced increase in incidence over time. Additionally, this explanation would not account for the differences in the rates of increase in IRs between gender, racial, and age-based subgroups.

One of the notable findings of our study was the fact that MMHN incidence is rising in nasal cavity lesions, but the age-adjusted IR for tumors outside of the nasal cavity has remained stable. This finding suggests that there is a specific etiologic agent contributing to nasal cavity melanomas that is either not present or does not have the same carcinogenic effects in other subsites. In a separate recently published SEER analysis, Jethanamest et al. demonstrated that nasal cavity melanomas are associated with superior survival compared to mucosal melanomas in other head and neck subsites, which lends further support to the hypothesis that nasal cavity melanomas are somehow biologically distinct from other MMHNs [27].

Of the patients identified in our SEER analysis from 1987 to 2009, patients with MMHN (*n* = 452) made up less than 0.5 percent of all patients with melanoma (*n* = 110, 537). Our findings confirm that MMHN is a rare malignancy. There was a slight predominance of female patients (52.4%) compared to male patients (47.6%). In contrast to our data, prior reports have described a modest male predominance in MMHN [28]. Given that our study is derived from a population-based dataset, our findings may reflect more accurately the true distribution of MMHN in the community. The most common site of disease in our study was the nasal cavity, comprising 52.4% of patients, and 72.6% of cases occurred in a sinonasal subsite. This finding is consistent with previous reports that have shown that the majority of cases of MMHN present in a sinonasal location [28].

Our study has several strengths. Primarily, the SEER data is population-based and is therefore highly generalizable. Second, by using the SEER data, we were able to identify patients diagnosed with MMHN over a long period of time who were diagnosed in multiple geographic regions of the United States. Limitations of our study include the potential for underreporting of minority data in the SEER registries, possible mistakes in coding, possible inconsistencies of coding across registries, and the relatively low number of cases. Additionally, the SEER data is limited in its ability to identify specific patient and disease factors that may contribute to the development of MMHN. These include (but are not limited to) family history, sun exposure history, occupational exposures, smoking history, and comorbid illnesses. Finally, as MMHN is often misclassified pathologically [29], it would be useful to have the ability to confirm each patient's pathologic diagnosis. Unfortunately, there is no mechanism within the SEER database for review of the original pathologic material. Overall, however, the SEER database is an excellent resource for reporting cancer trends over time, and we believe that our study forms a strong foundation for future work in this disease.

In summary, MMHN is a rare and often fatal malignancy that is increasing rapidly in incidence in the United States. The most profound increasing incidence is primarily in patients with nasal cavity tumors and in white women, with the most significant increases in incidence observed in white women ages 55 to 84. While MMHN and cutaneous melanoma do not appear to have a common etiologic basis, the rates of increase in the incidence of these diseases are similar. Further research on MMHN is needed, and we hope that our hypothesis-generating study will help to form a basis for future investigation that may elucidate the etiology and epidemiology of this aggressive disease.

## Figures and Tables

**Table 1:** Characteristics of patients with mucosal melanoma of the head and neck identified in SEER 9 from 1987 to 2009.

	# of patients	% of total
Sex		
Male	215	47.6%
Female	237	52.4%
Race		
White	383	84.7%
Black	24	5.3%
Other*	44	9.7%
Unknown	1	0.2%
Age		
0–19	0	0.0%
20–39	22	4.9%
40–54	50	11.1%
55–69	125	27.7%
70–84	190	42.0%
85+	65	14.4%
Site		
Nasal cavity	237	52.4%
Paranasal sinuses	89	19.7%
Middle ear	2	0.4%
*Total Sinonasal *	328	72.6%
Oral cavity	78	17.3%
Oropharynx	13	2.9%
Nasopharynx	20	4.4%
Parotid gland	13	2.9%
*Total Non-sinonasal *	124	27.4%

Total	452	

*Includes American Indian/Alaska Native and Asian/Pacific Islander.

**Table 2:** Total percent change (PC) and annual percent change (APC) for subgroups of patients with MMHN identified in SEER 9 registries from 1987 to 2009.

	PC (%)	APC (%)	*P* value^‡^
Site			
Sinonasal	135.3	2.7	<0.01
Nasal cavity	102.9	2.7	<0.01
Paranasal sinuses	286.6	2.3	0.19
Non-Sinonasal	−40.3	1.1	0.40
Sex			
Male	35.2	1.0	0.30
Female	45.5	3.4	<0.01
Age			
20–54	−42.1	—	—
55–84	184.4	2.9	<0.01
85+	−34.4	—	—
Sex and Race			
White male	12.6	0.6	0.52
White female	25.6	3.4	<0.01
White Females by Age			
Age 20–54	−80.2	—	—
Age 55–84	306.3	5.1	<0.01
Age 85+	−44.1	—	—

Blank fields indicate that the statistic could not be calculated due to insufficient patient numbers in the respective subgroup.

^‡^ *P* values refer to the likelihood that the APC is not different from zero by the weighted least squares method.
